# Time delay measurement in the frequency domain

**DOI:** 10.1107/S1600577515014095

**Published:** 2015-08-06

**Authors:** Stephen M. Durbin, Shih-Chieh Liu, Eric M. Dufresne, Yuelin Li, Haidan Wen

**Affiliations:** a Department of Physics and Astronomy, Purdue University, West Lafayette, IN 47907, USA; b Advanced Photon Source, Argonne National Laboratory, Argonne, IL 60439, USA

**Keywords:** pump–probe, X-ray synchrotron, ultrafast time resolution, RF frequency analysis

## Abstract

Pump–probe studies at synchrotrons using X-ray and laser pulses require accurate determination of the time delay between pulses. This becomes especially important when observing ultrafast responses with lifetimes approaching or even less than the X-ray pulse duration (∼100 ps). The standard approach of inspecting the time response of a detector sensitive to both types of pulses can have limitations due to dissimilar pulse profiles and other experimental factors. Here, a simple alternative is presented, where the frequency response of the detector is monitored *versus* time delay. Measurements readily demonstrate a time resolution of ∼1 ps. Improved precision is possible by simply extending the data acquisition time.

## Abstract 2

A simple frequency domain technique for determining the time delay between laser pump and X-ray probe pulses achieves 1 ps resolution even for ∼100 ps synchrotron pulses, permitting improved pump–probe characterization of ultrafast processes.

## 1. Introduction   

Dynamics of photoexcited states are probed at synchrotrons with pump–probe experiments using an optical laser pump pulse to excite a sample and a subsequent synchrotron pulse to probe it *via* diffraction, absorption or other techniques (Chen *et al.*, 2010 ▸). Excited-state lifetimes are deduced by comparing the synchrotron probe response to the time delay between the laser and synchrotron pulses. To investigate dynamics on short time scales near the ∼100 ps synchrotron pulse duration, it is crucial to have an accurate determination of the time delay. This is typically accomplished in the time domain, where a detector sensitive to both pulses produces an output signal permitting determination of the approximate time when the two pulses are coincident. Because the laser and synchrotron pulses have different pulse widths and shapes and are further convoluted with the detector response, there can be some arbitrariness in this determination. Here we report on an alternative approach in the frequency domain that may prove more robust in certain cases and allow for a more precise determination of time delay.

## 2. Methods and results   

The challenge of measuring the time delay between X-ray pump and optical probe pulses is closely related to the problem of synchronizing the two pulse trains. X-ray pulses are closely tied to the RF frequencies that control the storage ring at synchrotron sources. Subharmonics of the principal RF frequency can be used to drive the output of the pulsed laser system, thus synchronizing them to the X-ray pulses. Phase shifting the RF signal at the laser then changes the time delay between the synchronized X-ray and laser pulses (DeCamp *et al.*, 2005 ▸). Similar techniques have been developed for achieving femtosecond synchronization of an RF signal to a pulsed laser and between a pair of laser oscillators (Shelton *et al.*, 2002 ▸; Ma *et al.*, 2001 ▸; Kim *et al.*, 2004 ▸).

**Figure 1:**
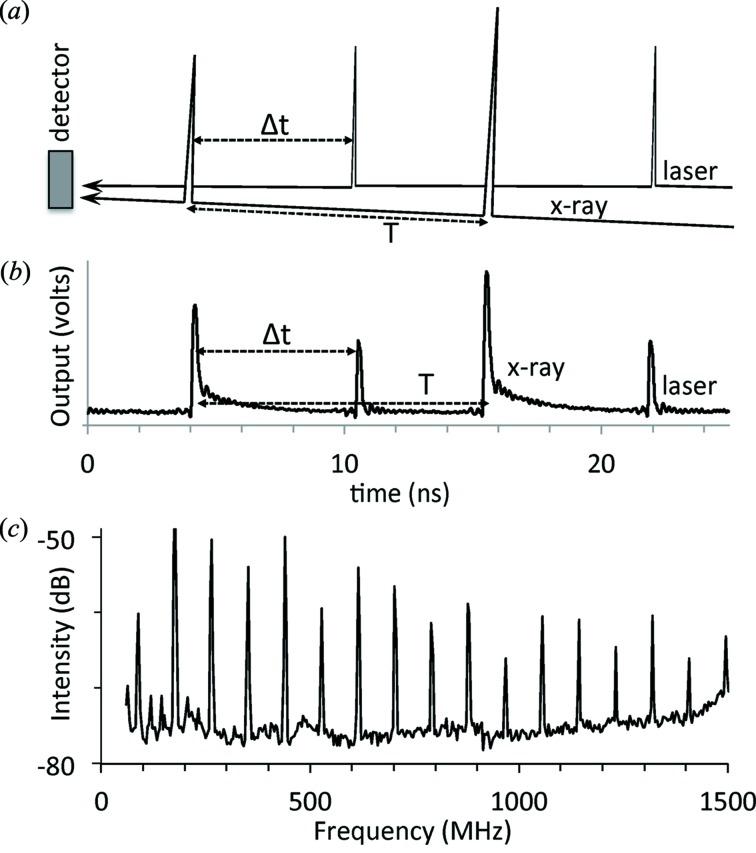
Laser and X-ray pulses in the time and frequency domains. (*a*) X-ray synchrotron pulses with period *T* and laser pulses with the identical period delayed by a time Δ*t* impinge on the same detector. (*b*) Time profile of the detector output, showing the sum of the two pulse trains. (*c*) RF spectrum analyzer output from the detector signal, showing the harmonics of the fundamental frequency. These peak intensities are a function of the time delay Δ*t*.

Frequency domain determination of the time delay simply requires sending the output of the detector to an RF spectrum analyzer. This converts the time domain signal into a series of peaks in the frequency domain, where each peak is at an integer multiple of a fundamental frequency. Consider having one laser pulse for each X-ray pulse, corresponding to the conditions of the data presented below. For symmetric fill patterns where the synchrotron pulses are equally spaced with time *T*, we denote the fundamental frequency as *f*_1_ = 1/*T*. Fig. 1(*a*) ▸ illustrates a stream of X-ray synchrotron pulses separated by *T* and a stream of laser pulses with the same period *T* striking the same detector; the laser pulses are delayed with respect to the X-ray pulses by a time Δ*t*.

**Figure 2:**
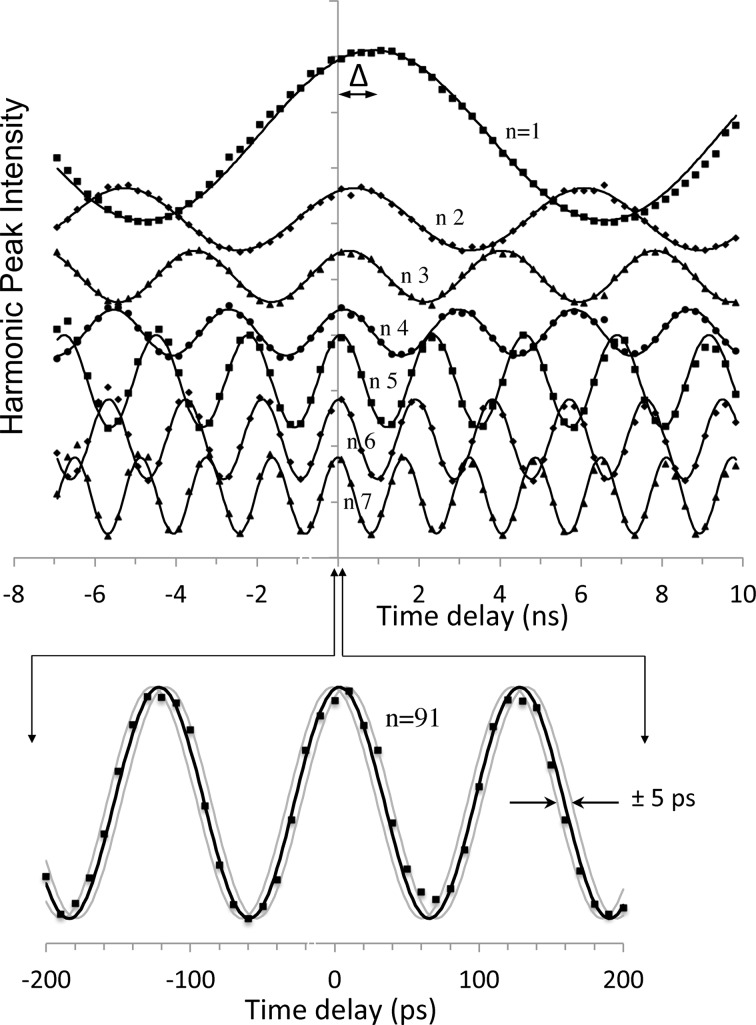
Phase dependence of the spectrum analyzer output. (Top) Peak intensities of the first seven harmonics *versus* time delay Δ*t*, plotted with a best-fit sine wave; curves are offset for clarity. Note that the peak of the fundamental is offset from zero by ∼0.8 ns, with rapidly decreasing offsets for the higher harmonics, due to the long tails of the detector pulses in the time domain (see text). (Bottom) Expanded plot of the 91st harmonic. The best-fit curve determines the coincidence time to better than 1 ps precision; curves shifted by ±5 ps are shown for comparison.

The detectors are typically some type of semiconductor diode or photoconductor with fast response times, and the output voltage profile recorded by an oscilloscope is illustrated in Fig. 1(*b*) ▸. The detector introduces some noise as well as a finite rise time and a slower fall time for the pulses. Sending the detector output instead to a spectrum analyzer produces an output like that shown in Fig. 1(*c*) ▸. Intensities of the frequency spectrum peaks measured *versus* the time delay Δ*t* reveal a strong sinusoidal dependence (Fig. 2 ▸).

Note that although the same fast diode detector is used for both the X-ray and the optical pulses, the physical interaction of X-ray and optical photons with the detector can be quite different. The optical photon simply promotes one valence electron into the conduction band whereas the X-ray process begins with deep core excitation and results in many electrons and holes. X-ray and laser photons may also have significantly different penetration depths into the semiconductor, as well as different noise characteristics. While these issues may affect the output temporal profiles, we find that frequency domain spectral analysis is largely insensitive to these differences, simplifying the requirements for both the detectors and the excitations sources amenable to this method.

The measurements shown in Fig. 2 ▸ were taken at the Sector 7-ID-C beamline of the Advanced Photon Source X-ray synchrotron running with 324 equally spaced bunches with a pulse frequency of *f*_1_ = 87.98 MHz (or *T* = 11.37 ns) (Dufresne *et al.*, 2010 ▸). The undulator output was monochromated to 12 keV and focused with Kirkpatrick–Baez mirrors to a ∼50 µm spot size. A Ti:sapphire laser oscillator produced 800 nm pulses of ∼100 fs duration that were focused to a similar spot size on the detector. Laser pulses were synchronized to the X-ray pulses by phase locking the fourth subharmonic of the 351.1 MHz RF signal that drives the electron bunches, which matches the pulse frequency *f*_1_ (Dufresne *et al.*, 2011 ▸). This signal controls the repetition rate of the laser, ensuring one laser pulse for each X-ray pulse. The time delay between X-ray and laser pulses is controlled by a delay generator coupled to a mechanical RF phase shifter, which adds a phase shift to a signal derived from the synchrotron RF; the laser pulse output is locked to this shifted signal. Time delay is set by computer control of the delay generator.

## 3. Analysis   

The nominal synchronization between X-ray and laser pulses due to the control electronics alone should be as low as 1 ps, but random contributions from such factors as temperature fluctuations yield an observed jitter of 10 ps or more. Frequency domain measurements are capable of higher temporal sensitivity, as noted below. While time domain spectra are broadened by random jitter, in the frequency domain random fluctuations tend to average out, allowing the average time delay between pulses to be determined with greater precision. This is a function of bandwidth setting of the spectrum analyzer, *i.e.* the effective signal averaging time.

Two different detectors were used and gave similar results. The first was a fast InGaAs diode that is routinely used for time domain determination of Δ*t* at this beamline (Landahl, 2004 ▸). The second was a coplanar stripline device on semi-insulating GaAs, a photoconductor whose response to X-rays and laser pulses has been reported on elsewhere (Durbin *et al.*, 2013 ▸). While the diode has a somewhat faster recovery time than the photoconductor, its frequency domain responses were similar. This reduced sensitivity to the response characteristics of a given detector demonstrates an advantage of the frequency domain method, which allows greater temporal resolution than would be expected from usual time response metrics. On the other hand, we do assume that the frequency response of a given detector to the X-ray and laser pulses are the same. If there is a significant difference due to very different penetration depths, path lengths, *etc*., then there could be a systematic offset on the apparent time delay between pulses.

The detector output was connected directly to an RF spectrum analyzer (either an HP E4411B for scans up to 1.5 GHz, or an HP EE4408 which can reach 26 GHz). Note that the basic function of this type of spectrum analyzer is to record a ‘frequency selective, peak responding’ voltage corresponding to ‘the r.m.s. value of a sine wave’ (Keysight Technologies, 2015 ▸). Each step in the data collection required setting the value of the time delay and recording a frequency spectrum. After all scans were taken, the peak heights of particular harmonics were plotted *versus* delay time. (This step-wise collection of data could easily be automated to speed up the process and reduce some possible uncertainties.)

Fig. 2 ▸ shows various scans taken with the photoconducting GaAs detector. A simple sinusoidal oscillation is seen for each harmonic, a phase sensitivity due to the interference of the detector signals generated by the laser and X-ray pulses. Writing the Fourier transform of the X-ray signal as
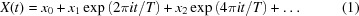
and the time-delayed laser signal as
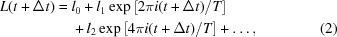
the *n*th term in the sum of the two functions yields
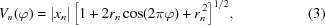
where *r*_*n*_ is the ratio of the amplitudes *l*_*n*_/*x*_*n*_, and φ = Δ*t*/*T*. This function is fit to the data, where the most important result is the value of time *t* for φ = 0, since this corresponds to the simultaneous arrival of both pulses.

Two important issues are highlighted in Fig. 2 ▸. First, it is seen that the maximum in the *n* = 1 harmonic does not occur at *t* = 0 but instead is shifted by Δ = 0.88 ns. Higher-order harmonics also exhibit shifts, but these decrease rapidly and are effectively gone at *n* = 7. The cause of these offsets is the asymmetry of the temporal response of the detector: the pulse turns on quickly but decays more slowly. (Dispersion was ruled out by observing the same shifts with a much longer coax cable between the detector and the spectrum analyzer.) The lowest-order Fourier component will be shifted towards later times because of the tail in the peak, whereas the higher-order components are controlled more by the sharp initial rise. The observed shifts are consistent with the observed exponential decay of several hundred picoseconds for the GaAs stripline detector used here.

The second key issue is: how precisely can the time delay be determined? Clearly one needs to go to the highest-order harmonic that still has good signal-to-noise; Fig. 2 ▸ (bottom) shows a fit to data taken at *n* = 91 (or a frequency of ∼8 GHz). This best fit to these data was obtained with a non-linear regression analysis that determined an uncertainty of less than 1 ps. This could be made arbitrarily smaller by acquiring data over a larger number of periods, at least to the extent that drifts in the laser and X-ray pulse power and positions are avoided. The time delay determined from any given frequency component is only known modulo the period of that component; measurements at multiple frequencies readily remove that ambiguity. Note in Fig. 2 ▸ that the phase shifts due to asymmetries in the temporal profiles have disappeared after very few harmonics, allowing a well defined time delay to be determined.

## 4. Conclusion   

We have demonstrated a simple approach for determining the time delay between synchrotron X-ray and laser pulses for pump–probe applications, requiring only the addition of an RF spectrum analyzer. Time delays were determined with a precision of only 1 ps, even for X-ray pulses with ∼100 ps widths. This precision could be readily improved by extending the data acquisition to cover a greater number of periods of the highest detectable harmonic. The accuracy depends in principle on the detector having the same frequency response to both X-rays and optical photons. This new phase-sensitive frequency domain approach may be more robust compared with traditional time-domain measurements for the study of dynamics with picosecond lifetimes.
